# Selenium preserves mitochondrial function, stimulates mitochondrial biogenesis, and reduces infarct volume after focal cerebral ischemia

**DOI:** 10.1186/1471-2202-13-79

**Published:** 2012-07-09

**Authors:** Suresh L Mehta, Santosh Kumari, Natalia Mendelev, P Andy Li

**Affiliations:** 1 Department of Pharmaceutical Sciences, Biomanufacturing Research Institute and Technology Enterprise (BRITE), North Carolina Central University, BRITE Building 2025, 302 East Lawson Street, Durham, NC, 27707, USA

**Keywords:** Cerebral ischemia, Glutamate, Hypoxia, Mitochondrial biogenesis, Respiratory complexes, Selenium

## Abstract

**Background:**

Mitochondrial dysfunction is one of the major events responsible for activation of neuronal cell death pathways during cerebral ischemia. Trace element selenium has been shown to protect neurons in various diseases conditions. Present study is conducted to demonstrate that selenium preserves mitochondrial functional performance, activates mitochondrial biogenesis and prevents hypoxic/ischemic cell damage.

**Results:**

The study conducted on HT22 cells exposed to glutamate or hypoxia and mice subjected to 60-min focal cerebral ischemia revealed that selenium (100 nM) pretreatment (24 h) significantly attenuated cell death induced by either glutamate toxicity or hypoxia. The protective effects were associated with reduction of glutamate and hypoxia-induced ROS production and alleviation of hypoxia-induced suppression of mitochondrial respiratory complex activities. The animal studies demonstrated that selenite pretreatment (0.2 mg/kg i.p. once a day for 7 days) ameliorated cerebral infarct volume and reduced DNA oxidation. Furthermore, selenite increased protein levels of peroxisome proliferator-activated receptor-γ coactivator 1alpha (PGC-1α) and nuclear respiratory factor 1 (NRF1), two key nuclear factors that regulate mitochondrial biogenesis. Finally, selenite normalized the ischemia-induced activation of Beclin 1 and microtubule-associated protein 1 light chain 3-II (LC3-II), markers for autophagy.

**Conclusions:**

These results suggest that selenium protects neurons against hypoxic/ischemic damage by reducing oxidative stress, restoring mitochondrial functional activities and stimulating mitochondrial biogenesis.

## Background

Cerebral ischemia/stroke, like other neurodegenerative diseases, increases destruction of neurons by activating cascade of events such as glutamate-induced excitotoxicity, oxidative stress, protein synthesis inhibition, apoptosis, and autophagy etc. Brain is very susceptible to these events in particular to oxidative stress owing to its high metabolic rate, presence of high content of polyunsaturated fatty acids and postmitotic nature of neurons than most other organs. Increased reactive oxygen species (ROS) production disrupts antioxidant defense and directly impairs mitochondrial homeostasis and energy production [1-3].

Trace element selenium (Se) has been shown to be important to human health and associated with several human diseases including Keshan disease, cancer, virus infections, male infertility, abnormalities in immune responses, metabolic and neurological disturbances and developmental delays [4]. Selenium is an essential component of the rare amino acid selenocysteine (Sec) and is incorporated at the catalytic site of various selenium-dependant enzymes such as glutathione peroxidase (GPx), thioredoxin reductases, and one methionine-sulfoxide-reductase. These selenoenzymes play important roles in regulating metabolic activity, immune function, antioxidant defense and intracellular redox regulation and modulation [5,6]. Decreased activities of these selenoenzymes caused either by depletion/insufficient levels of Se or mutation results in exacerbation of neuronal loss and dysfunction. Likewise, genetic inactivation of all selenoproteins in neurons leads to progressive neurodegeneration [7]. Selenium supplementation-dependent increases in selenoenzyme activity or overexpression of selenoenzymes, in contrast, ameliorates outcome caused by endogenous or exogenous stress, hypoxia, trauma and other neurodegenerative conditions including cerebral stroke [8-14]. Moreover, selenium modulates several cell signaling pathways, including activating the mitogen-activated protein kinase (MAPK), phosphotidylinositol 3-kinase (PI3K)-Akt, and NF-kB pathways [15-17].

Previous studies have demonstrated that selenium supplementation ameliorates hypoxia/ischemia-induced neuronal death *in vitro* and *in vivo*[18,19]. However, it is not known whether selenium is capable of preserving mitochondrial function *in vitro* after glutamate exposure and whether selenium neuroprotective effect is associated with activations of mitochondrial biogenesis regulators and autophagy in mice that are subjected to a transient focal cerebral ischemia. The present study investigates the neuroprotective effect of selenium pretreatment on glutamate toxicity, hypoxia and ischemic brain damage, and its association to mitochondrial function. In addition, we assessed the influence of selenium on the protein levels of two nuclear transcription factors, nuclear respiratory factor-1 (NRF1) and peroxisome proliferator-activated receptor-γ coactivator-1 alpha (PGC-1α), which regulates mitochondrial biogenesis. Furthermore, we examined autophagy status by measuring protein levels of Beclin 1 and microtubule-associated protein 1 light chain 3 (LC3). We found that selenium pretreatment increased cell viability, decreased cell death, lowered ROS production and improved mitochondrial functional performance after glutamate exposure and/or hypoxia. The effects of selenium are well translated in animal stroke model. Thus, selenium reduced infarct volume and suppressed oxidative DNA damage. In addition, selenium pretreatment increased levels of mitochondrial biogenesis regulators and reduced level of autophagy modulators.

## Methods

### Cell culture, treatment and harvest

Murine hippocampal neuronal HT22 cells were maintained in Dulbecco's Modified Eagle Medium (DMEM)/F12 containing 10% fetal bovine serum (FBS), 2 mM glutamine, and 200 mM streptomycin/penicillin (Invitrogen) and then maintained at 90–95% relative humidity in 5% CO_2_ at 37 °C. The culture medium was renewed every 3 days. Cells were treated with 100 nM sodium selenite (Na_2_SeO_3_; Sigma, cat. 214485) prepared in phosphate buffered saline (PBS) with 1% BSA; pH 7.6 for 24 h prior exposure to glutamate or hypoxia based on previous study [20]. Glutamate toxicity was induced by incubating the cells with 4 mM glutamate and effects were tested 24 h after exposure. Hypoxia was produced by bubbling DMEM media with N_2_ until oxygen falls below 5% of detectable level in an oxygraph glass chamber (Oroboros Instruments, Austria). The final oxygen content in the chamber was maintained at 2.5 ± 1.0 nmol/ml [21]. Oxygraph allows continuous monitoring of oxygen level at very high resolution. After 10 h of hypoxia, cells were plated and transferred to incubator maintained at 90–95% relative humidity in 5% CO_2_ at 37 °C to allow reoxygenation. All experiments were performed in triplicate with at least 2 repetitions.

### Determination of ROS and mitochondrial membrane potential

Intracellular ROS (superoxide anion) production and mitochondrial membrane potential were measured using dihydroethidium (DHE) and tetramethylrhodamine, methyl ester (TMRM) respectively in selenium-pretreated cells exposed to glutamate (4 mM) or hypoxia (10 h). ROS production was measured 24 h or 10 h after glutamate or hypoxia exposure respectively. Briefly, cells (2x10^6^/ml) were incubated with the DHE (2.5 μM) or TMRM (100 nM) for 30 min at 37 °C. Cells were washed, resuspended in PBS and analyzed for fluorescence intensity using Fluoromax-4 spectroflorometer (HORIBA Jobin Yvon Inc, Edison, NJ) at the excitation and emission wavelengths of 480 nm and 590 nm for ROS and at the excitation 530 nm and emission 573 nm for mitochondrial membrane potential respectively. The florescence recorded was represented as relative intensity (%).

### Measurements of mitochondrial respiration and complex activities

Polarographic respiration measurement at different complexes was performed in the presence of 0.5 M ADP to analyze activity of each complex using multiple substrate-inhibition protocol [22]. Measurement was done using a high resolution respirometer (Oxygraph, Oroboros Instrument) equipped with a peltier thermostat and electromagnetic stirrer at 37 °C. Briefly, digitonin-permeabilized normal and selenium pretreated HT22 cells (1x10^7^) were incubated in 2 ml mitochondrial respiration medium MiR05 (110 mM sucrose, 0.5 mM EGTA, 3.0 mM MgCl_2_, 60 mM K-lactobionate, 10 mM KH_2_PO_4_, 20 mM Taurine, 20 mM HEPES, 1.0 g/l BSA, pH 7.1) in a glass chamber. Hypoxia was produced by bubbling medium with N_2_ until oxygen falls below 5% [21] of detectable level (Oroboros Instruments, Austria). After 15 min of hypoxia, chamber was opened to allow reoxygenation. The following substrates and inhibitors were used for complex I; glutamate (10 mM), malate (5 mM) and rotenone (0.5 μM); for complex II + III: succinate (10 mM) and antimycin A (2.5 μM), and for complex IV: N,N,N’,N’-tetramethyl-p-phenylenediamine dihydrochloride (TMPD, 0.5 mM), ascorbate (2 mM) and potassium cyanide (KCN, 1.0 mM). The integrity of the outer mitochondrial membrane following digitonin permeabilization was confirmed with cytochrome C (10 mM). Two hypoxic schemes were used in the study: 10 h hypoxia in non-permeated and 15 min hypoxia in digitonin permeabilized cells. Hypoxia of 10 h was sufficient to induce cell death in non-permeabilized cells whereas permeabilized cells were not able to endure 10 h of hypoxia. Therefore, hypoxic period was reduced to 15 min according to our pilot study.

### Experimental animals and groups

A total of 43 male C57BL/6 J mice weighing 25-28 g were used for the experiments. Animals were pretreated with saline or sodium selenite (0.2 mg/kg, i.p.) for 7 days followed by induction of cerebral ischemia. Animals were subjected to 1 h of ischemia and 5- and 24 h of recirculation. Both selenium and saline treated mice (n = 20 in each group) were divided into sham-operated (7 each), 1 h of ischemia plus 5- (4 each) and 24 h (9 each) of recirculation. Three mice were excluded due to unsuccessful occlusion. For surgery, the animals were fasted overnight with free access to water. Anesthesia was induced by 3% isoflurane with N_2_O/O_2_ (70/30) and maintained at 1.5% during the operation. The operative animal procedures were approved by the Institutional Animal Care and Use Committee (IACUC) at North Carolina Central University.

### Ischemic model

Transient middle cerebral artery occlusion (MCAO) was induced by the intraluminal filament technique as described previously [23]. Briefly, the right common carotid artery (CCA), internal carotid artery (ICA) and external carotid artery (ECA) were exposed through a midline incision. A filament coated with silicon rubber (diameter of 0.21 ± 0.02 mm) was inserted into the ICA through the CCA to occlude the origin of the middle cerebral artery (MCA). Filament was withdrawn to allow recirculation after 1 h of MCAO. The core temperature during surgery was maintained at 37 ± 0.5°C by a heating blanket. After the occlusion the mice were examined and only animals with neurological signs of diminished resistance to lateral push, walking to the left after being pulled backwards by the tail, or with spontaneous contralateral circling were included in the study [24]. Upon predetermined end points (5- and 24 h), animals were euthanized and the brains were either perfusion fixed with 4% paraformaldehyde or frozen in liquid nitrogen for later studies.

### Measurements of infarct volume

Paraformaldehyde perfusion-fixed brains were sectioned with vibratome (30 μm thick) and preserved in antifreeze solution at −20°C until use. Seven coronal brain sections collected from sham control (n = 3 each group) and 24 h recirculation (n = 5 each group) from bregma 2.20 mm to −4.16 mm were selected and stained with propidium iodide (PI) (Vector Laboratories Inc., Burlingame, CA) to view nuclear morphology. Neuronal morphology was also confirmed with NeuN staining by using anti-NeuN antibody (1:300, mouse monoclonal; Chemicon, Temecula, CA). Images were captured, areas with condensed nuclei were outlined, and infarct volume was calculated using the NIH ImageJ software. As described previously, histo-mophorlogical studies give priority to detect ischemic brain damage over 2,3,5-triphenyltetrazolium chloride (TTC) staining [25].

### Fluoro-Jade B staining

Fluoro-Jade B staining was used to confirm neurodegeneration on brain section [26]. Briefly, vibratome brain sections were mounted; air dried on glass slides and briefly immersed in descending concentration of ethanol and distilled water. The slides were then transferred to a solution of 0.06% potassium permanganate (KMnO_4_) for 15 min and rinsed with water. Slides were then immersed in 0.001% Fluoro-Jade B solution (Millipore) in 0.1% acetic acid for 30 min and rinsed with water followed by coverslipping with mounting media (Vector Laboratories Inc. Burlingame CA). Double labeling with NeuN was performed as per the protocol of the manufacturer.

### Detection of oxidative DNA damage

Vibratome brain sections (30 μm) of animals subjected to 1 h of ischemia and 24 h of recirculation were used to detect oxidative DNA damage using anti-8-hydroxy-2-deoxyguanosine (anti-8-OHdG, 1:1000; ab62623 Abcam, Cambridge, MA). Sections were incubated overnight with primary antibody against 8-OHdG. These sections were then incubated with anti-rabbit IgG (Alexa Fluor 488 1:500, Invitrogen) secondary antibody. The sections were mounted with Vectashield mounting medium containing DAPI (Vector Laboratories Inc. Burlingame CA) and scanned using a Nikon laser-scanning confocal microscope at 400X final magnification. Three microscopic fields per section from dorso-lateral striatum and the overlying cortex were captured for analysis.

### Western blot analysis

Ischemic cortical area (penumbra) was dissected (n = 4 per subgroup) and homogenized. Nuclear and cytosolic fractions were extracted by a series of centrifugations [27]. Protein concentration was determined by the Bradford method (Bio-Rad) and 20 μg protein of each sample was loaded and run in 4-12% NuPAGE gel (Invitrogen). Following electrophoresis and transfer, the membranes were incubated overnight with primary antibodies against NRF1 (1:500, sc-23624, Santa Cruz), PGC-1α (1:500, ST1202, Chemicon), Beclin 1 (1:500, sc-11427, Santa Cruz), LC-3 (1 μg/ml, M115-3 MBL), histone H3 (1:2000, 05–928 Millipore) or β-actin (1:1000, Santa Cruz). The membranes were incubated with horseradish peroxidase-conjugated secondary antibodies for 1 h. The immunoblots were developed using the Pierce ECL Western blotting substrate (Thermo Scientific, Rockford IL). The protein bands of β-actin or histone 3 (H3) were used as internal loading controls. The results were presented as ratios of respective proteins and loading control.

### Immunohistochemistry

Brain vibratome sections were washed in TBS/0.1% TX100 (TBST). The nonspecific binding sites were blocked with 5% donkey serum in TBST and incubated overnight with primary antibody against LC-3 (1 μg/ml, M115-3 MBL) and phospho-dynamin-related protein 1 (pDrp1, 1:1600, Cell Signaling Technology). pDrp1 is used to demarcate mitochondrial fragmentation [28] and to determine whether LC3 staining overlaps with pDrp1 positive mitochondria. These sections were then incubated with secondary antibody (Alexa Fluor 488/568 1:500, Invitrogen). The sections were mounted with mounting medium containing DAPI and scanned under Nikon laser-scanning confocal microscope at 400X final magnification. Three microscopic fields per section were captured and analyzed for positive changes.

### Statistics

Statistical analysis for significant changes was performed with the GraphPad Prism statistical software. Multiple comparisons were analyzed with two-way ANOVA followed by Scheffe’s test. All data are given as means ± SD. *p < 0.05, **p < 0.01, ***p < 0.001 compared with controls and #p < 0.05 & ##p < 0.01, ##p < 0.001 when comparison was made between respective groups.

## Results

### Selenium prevents glutamate and hypoxia-induced cell death

**Figure 1:**
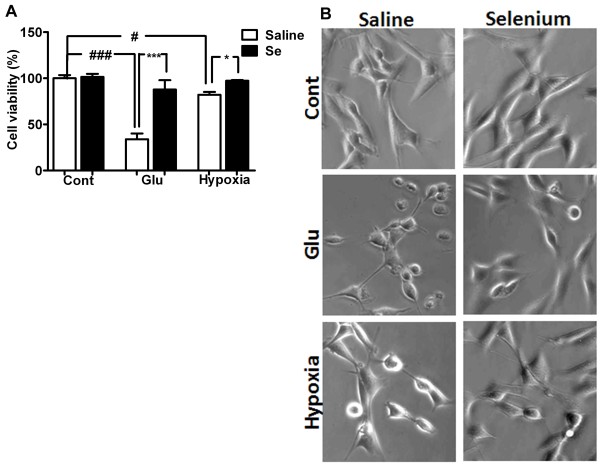
**Neuroprotective effect of selenium pretreatment against glutamate toxicity and hypoxia. A**. Effect of selenium pretreatment on glutamate toxicity and hypoxic cell death. Cell viability was measured 24 h or 10/12 h after glutamate and hypoxia/reoxygenation respectively. Selenium pretreatment significantly improved cell survival from glutamate toxicity (4 mM) and hypoxia/reoxygenation (10/12 h). **B**. Representative micrograph of HT22 cells exposed to glutamate (4 mM) and hypoxia/reoxygenation (10/12 h) with or without selenium pretreatment. Cell viability was calculated using MTT assay and relative cell viability was expressed as % of control set at 100%. Data represents means ± SD of 4 experiments conducted in triplicate. *P < 0.05, ***P < 0.001 *vs.* control and #p < 0.05, ###p < 0.001 *vs.* respective group. Se = selenium, Cont = control and Glu = glutamate.

Hippocampal HT22 neuronal cells were pretreated with selenium in the form of selenite (Na_2_SeO_3_) 24 h prior to glutamate or hypoxia exposure. Selenite concentration about 100 nM, which is within the physiological range [20], has no effect on cell viability (Figure 1A &1B). Cells exposed to 4 mM of glutamate for 24 h reduced cell survival by > 65%. Selenium pretreatment prevented glutamate-induced cell death and increased cell survival.

Similarly, hypoxia (10 h) reduced HT22 neuronal cell survival to 82% after 12 h of reoxygenation. Interestingly, selenium pretreatment improved cell survival and prevented cells death in HT22 neuronal cells (Figure 1A &1B). Therefore, cell survival increased from 82 to 95% after 12 h of reoxygenation.

### Selenium pretreatment reduces glutamate-induced ROS production and preserves mitochondrial membrane potential

**Figure 2:**
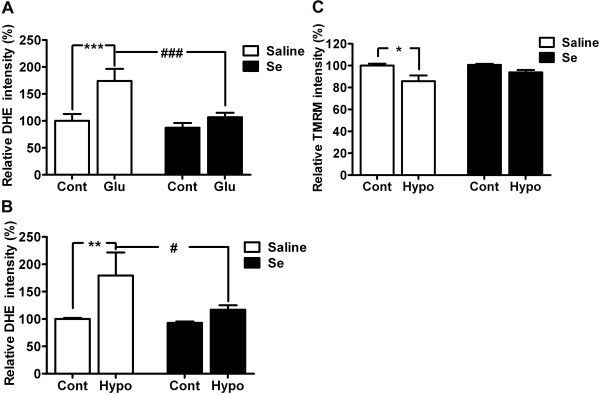
**Selenium pretreatment attenuates ROS production and maintains mitochondrial membrane potential.** ROS production was attenuated by selenium pretreatment following glutamate (**A**) or hypoxic exposure (**B**). ROS production was measured in the form of superoxide anion (which is one component of total ROS) using Dihydroethidium (DHE), after 24 h of glutamate or 10 h of hypoxia exposure in HT22 cell pretreated with selenium. **C**. Selenium pretreatment prevented the fall in mitochondrial membrane potential following hypoxic exposure. The results were expressed as relative DHE or TMRM intensity % of control set at 100% and represented as means ± SD. *P < 0.05, **P < 0.01 & ***P < 0.001 *vs.* control, and #*P* < 0.05 & ###p < 0.001 *vs.* respective group. Se = selenium, Cont = control, Hypo = hypoxia and Glu = glutamate.

To determine whether protective effect shown by selenium is associated with its antioxidant property, we tested the effect of selenium on ROS production. ROS were measured in the form of superoxide anions following glutamate or hypoxia exposure. As shown in Figure 2A, glutamate treatment significantly (p < 0.01) increased ROS levels in HT22 cells. The increased production of ROS is inversely correlated with cell viability (Figure 1). Interestingly, as hypothesized, selenium pretreatment not only lowered ROS levels in control but also significantly (p < 0.05) reduced ROS level in HT22 cells treated with glutamate. Similar results were also observed with hypoxia, where hypoxia significantly (p < 0.01) increased ROS levels and selenium pretreatment (p < 0.05) reversed this trend towards normal level (Figure 2B).

To further confirm the beneficial role of selenium, we measured mitochondrial membrane potential in cells treated with selenium and exposed to hypoxia (10 h) and reoxygenation (1 h). The result revealed that hypoxia affected mitochondrial function and significantly (p < 0.05) lowered mitochondrial membrane potential. Interestingly, selenium pretreatment preserved the mitochondrial membrane potential and thereby prevented the potential fall following hypoxic exposure.

### Selenium preserves mitochondrial respiration and complex activities

**Figure 3:**
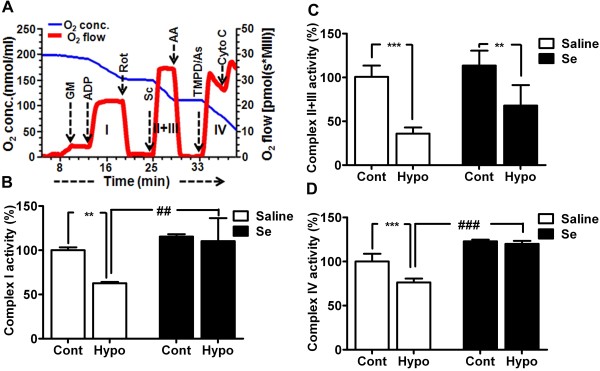
**Selenium pretreatment preserves mitochondrial respiratory chain complex activities following hypoxia. A**. A typical recording of oxygen consumption of digitonin permeabilized cells showing oxygen concentration (blue) and respiration (oxygen flux, red) using multiple substrates-inhibitor analysis. The activity of each complex was calculated from the difference in reduction in oxygen content in the presence of specific substrate and specific inhibitor. **B-D**. Activity of each mitochondrial complex (1, II, +III and IV) indicates the inhibition of hypoxia-suppressed complex activities by selenium pretreatment. Data represents means ± SD. **P < 0.01, ***P < 0.001 *vs.* control and ##p < 0.01, ###p < 0.001 *vs.* respective group. Se = selenium, Cont = control and Hypo = hypoxia. AA, antimycin A; As, ascorbate; Cyto C, cytochrome c; GM, glutamate and malate; Rot, rotenone; Sc, succinate; TMPD, N,N,N’,N’-tetramethyl-p-phenylenediamine dihydrochloride.

To determine whether the beneficial effect shown by selenium is mediated through mitochondria, we tested mitochondrial functional performance following hypoxia by measuring oxygen utilization using complex specific substrates (Figure 3A). We then calculated the activities of each mitochondrial respiratory complex from the difference in oxygen content reduction in the presence of specific inhibitor (Figure 3B-3D). As shown in Figure 3B-3D, hypoxia significantly decreased the activity of complex I, II + III and IV by 37 (p < 0.01), 65 (p < 0.001), and 24% (p < 0.01), respectively, as compared to control. Interestingly, selenium pretreatment slightly increased the activities of these complexes at basal level. Compared to selenium pretreated control, the complex I, II + III and IV activities in selenium-treated hypoxia model only reduced by 5, 45 and 3%, respectively; indicating that selenium pretreatment alleviated the effect of hypoxia on mitochondrial complexes. Therefore, the activities were either brought back to normal level (complex I and IV) or significantly improved (Complex II + III) by selenium as compared to non-Se-treated cells.

### Selenium pretreatment reduces ischemic brain damage

**Figure 4:**
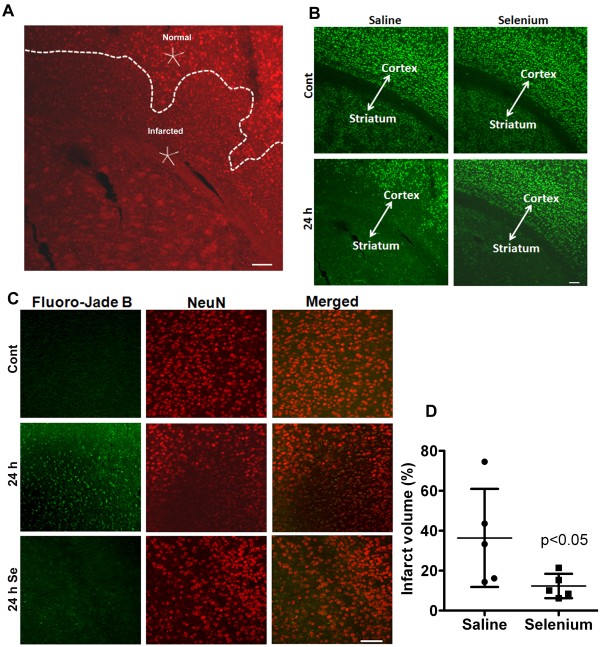
**Selenium pretreatment reduces ischemic brain damage after one hour of cerebral ischemia followed by recirculation. A**. Propidium iodide nuclear staining depicts the damaged area in contrast to the intact neighboring tissue. **B**. Microphotographs of NeuN immunostaining shows the neuronal population in control and ischemic brain area of selenium and saline pretreated mouse. Cerebral ischemia caused neuronal degeneration at 24 h of recirculation in striatum and some part of overlying cortex in saline treated mice. Selenium pretreatment, in contrast, restricted the neuronal damage to only striatal area of the brain after 24 h of recirculation. **C**. Fluoro-Jade B (green) and NeuN (red) staining confirm the loss of NeuN staining is associated with neurodegeneration. **D**. Graph shows infarct volume measured after 24 h of recirculation in both saline and selenium pretreated groups. Selenium pretreatment significantly decreased (*p* < 0.05) infarct volume at 24 h of recirculation. Data represents mean ± SD. *P < 0.05. Se = selenium and Cont = control. Bar = 100 μm.

To finally determine whether the protective effects of selenium observed in *in vitro* studies can be translated to an *in vivo* cerebral stroke model, we treated mice with selenium for 7 days prior inducing transient focal ischemia. We found that cerebral ischemia induced brain damage in animals subjected to 1 h of MCAO and 24 h of recirculation. Brain damage analyzed with propidium iodide (PI) staining clearly distinguished the infarct area from the healthy neighboring tissue (Figure 4A). Infarct area displayed phenotypic differences in the form of severely condensed nuclei in contrast to smooth rounded nuclear staining in the non-damaged area. These changes were further confirmed by anti-NeuN and Fluoro-Jade B stainings (Figure 4B &4C). The result revealed the loss of NeuN staining and cellular density in the ischemic side of the brain. Loss of NeuN staining was associated with neurodegeneration as revealed by Fluoro-Jade B staining, suggesting that neurons were greatly affected morphologically and spatially following cerebral ischemia in saline treated mice. Interestingly, selenium pretreatment prevented neuronal loss as revealed by preserved anti-NeuN staining and negative Fluoro-Jade B staining.

Measurements of infarct volume using anti-NeuN stained sections revealed that 1 h of MCAO resulted in damage to nearly one third of the ipsilateral hemisphere comprised by striatum and some part of overlying cortex at 24 h of recovery. In contrast, selenium pretreatment significantly (p < 0.01) reduced the brain damage. Therefore the infarct volume reduced from 36.4 ± 24.5 to 11.6 ± 5.0% in selenium treated mice as compared to saline control at 24 h of recovery (Figure 4D). These results clearly indicate the positive role of selenium in neuroprotection.

### Selenium pretreatment prevents cerebral ischemia-induced oxidative DNA damage

**Figure 5:**
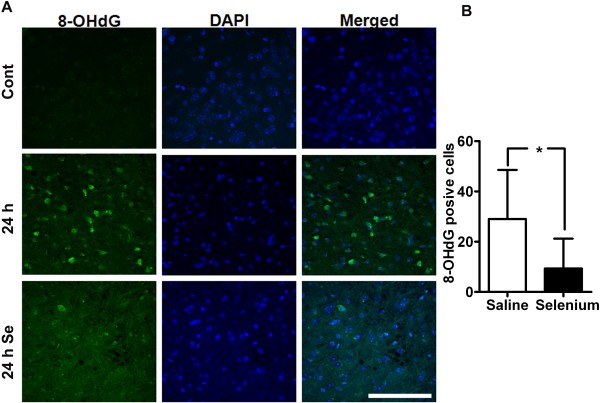
**Selenium reduces DNA oxidatioin. A**. A set of representative anti-8-OHdG immunofluorescent stained images (green) shows decline in 8-OHdG fluorescence signal at 24 h of recirculation in selenium pretreated as compared to saline treated. Blue color (DAPI) denotes nuclei. **B**. Bar graph shows the number of 8-OHdG positive cells per section in the cortex area of the brain. Data are presented as mean ± SD. *P < 0.05. Se = selenium and Cont = control. Bar = 100 μm.

As shown in Figure 2, selenium significantly reduced ROS level after 24 h of glutamate exposure. Therefore, we determined to study whether cerebral ischemia induces oxidative DNA damage and whether selenium has antagonistic effect on DNA oxidation. Very weak 8-OHdG immunoreactivity was observed in sham-operated animals (Figure 5A). After 24 h of recirculation, 8-OHdG immunoreactivity was clearly increased and presented in the nuclei of saline treated mice. In contrast, selenium pretreatment significantly reduced the number of 8-OHdG immunoreactive cells after 24 h of reperfusion as compared to saline treated mice (Figure 5B), indicating that antioxidant property shown by selenium may be associated with preventing DNA from being oxidized.

### Selenium pretreatment stimulates mitochondrial biogenesis regulators

**Figure 6:**
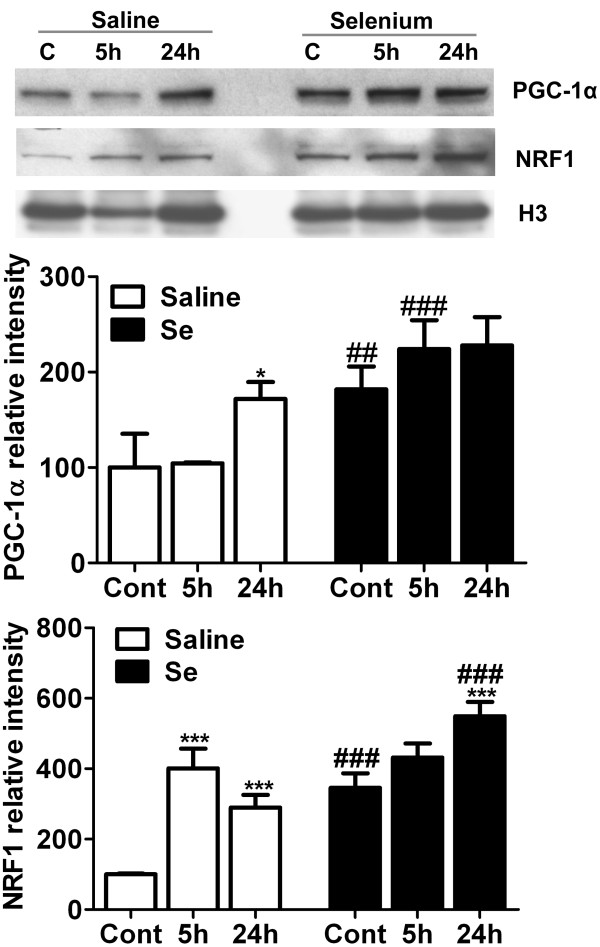
**Selenium pretreatment increases protein levels of mitochondrial biogenesis markers, PGC-1α and NRF1.** Representative Western blot and analysis of PGC-1α and NRF1 in control, 5- and 24 h of recirculation in saline and selenium pretreated groups (n = 4 each group). The results were normalized as relative % of control set at 100%. Data represents mean ± SD. *p < 0.05, and ***p < 0.001 *vs* control and ##p < 0.01, ###p < 0.001 *vs.* respective time point. Se = selenium and Cont = control.

Selenium pretreatment increases mitochondrial biogenesis *in vitro* (Li and collegues, communicated data). Therefore, we explored if selenium pretreatment could increase the levels of mitochondrial biogenesis regulators, NRF1 and PGC-1α, in *in vivo* stroke model. As shown in Figure 6, cerebral ischemia significantly increased the protein levels of PGC-1α in saline treated animals at 24 h (p < 0.05) of recirculation. Selenium pretreatment further increased PGC-1α at control levels and each time points of recirculation. Therefore, the increase was significant in control and at 5 h of recirculation animals as compared to respective time points of non-treated group.

Similarly, NRF1, a downstream transcription factor of PGC-1α, is significantly increased at 5- (p < 0.001) and 24 h (p < 0.001) of recirculation in mice treated with saline. Selenium pretreatment further increased the protein level of NRF1 at control and each time points of recirculation and the increase reached to significant level at 24 h (p < 0.001) of recirculation. Therefore, NRF1 significantly increased at control (p < 0.001) and 24 h (p < 0.001) in selenium pretreated group as compared to respective time points in saline treated ones. These results suggest that beneficial effect of selenium may be mediated through increasing mitochondrial protein synthesis and biogenesis.

### Selenium treatment normalizes ischemia-activated autophagy

**Figure 7:**
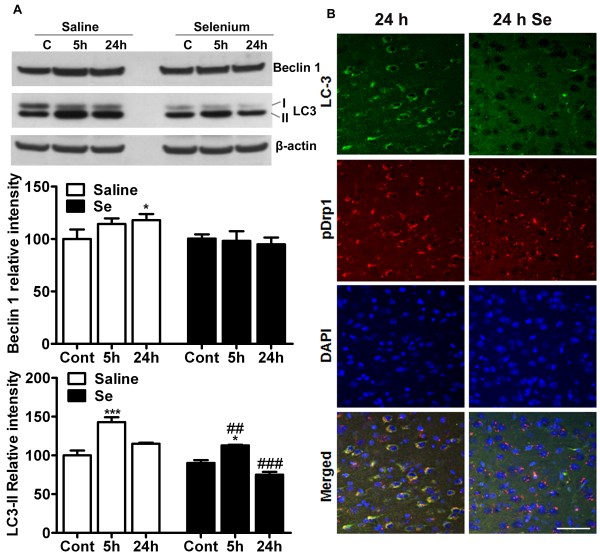
**Selenium pretreatment alters autophagy activation following cerebral ischemia. A**. Representative Western blot and analysis of autophagy markers Beclin 1 and LC3 shows that selenium pretreatment lowers that intensity Beclin 1 and LC3-II as compared to saline treatment. **B**. Immunolocalization of LC3 (green) and pDrp1 (red) indicates that cerebral ischemia leads to conversion of LC3-I (diffused) to LC3-II (punctated). Punctated LC3 pattern overlaps with pDrp1 staining. Selenium pretreatment, on contrary, decreases LC3-II (punctated) staining. The western results were normalized as relative % of control set at 100% (n = 4 each group). Data represents mean ± SD. *p < 0.05, and ***P < 0.001 *vs* control and ##p < 0.01, ###p < 0.001 *vs.* respective time point. Se = selenium and Cont = control. Bar = 100 μm.

Cerebral ischemia has been shown to activate autophagy, a main process involved in clearance of damaged organelles and debris. The activation of autophagy can be detected by measurements of Beclin 1 and LC3-II. Therefore, we examined whether selenium treatment affects the levels of Beclin 1 and LC3-II following focal cerebral ischemia. Western analysis of Beclin 1 in cytosolic fraction revealed that Beclin 1 level increased at 5- and peaked at 24 h in saline treated animals (Figure 7A). The increase at 24 h reached to significant level as compared to control. In selenium pretreated animals, the protein levels of Beclin 1 were either remained unaffected or showed slight decrease with increasing time of recirculation. Therefore, Beclin 1 levels remained close to baseline as compared to saline treated mice.

LC3, another marker of autophagy, is synthesized as pro-LC3 and then cleaved by Atg4 protease to LC3-I. Upon activation of autophagy, LC3-I is conjugated with phosphatidylethanolamine to form LC3-II, which become structural component of autophagosomes. As shown in Figure 7A, LC3-II levels significantly increased after 5 h (p < 0.001) of recovery and then declined to near baseline level at 24 h in saline treated animals. Similar trend was also observed in selenium pretreated animals, however, LC3-II level was significantly lower at 5- (p < 0.001) and 24 h (p < 0.001) of recirculation as compared to saline-treated animals with respective time points of recirculation.

These results were further confirmed with immunolocalization of LC3 and pDrp1 (Figure 7B). Cerebral ischemia increased LC3 staining as compared to control. Staining also shows the punctated pattern, which indicates the conversion of LC3-I to II following cerebral ischemia. Punctated LC3 staining overlapped with pDrp1 staining. Drp1, when phosphorylated, leads to mitochondrial fragmentation and fragmented mitochondrial could lead to increased cellular damage. Therefore, in the present study the colocalization of pDrp1 and LC3 indicates that fragmented mitochondria may be cleared up by autophagy dependant mechanisms. Interestingly, as shown in Figure 7A, selenium pretreatment reduced LC3 staining following cerebral ischemia, suggesting that cerebral ischemia activates autophagy in order to clear the damaged organelles and debris. In contrast, selenium pretreatment reduces ischemic brain damage and thereby lowers autophagy activation.

## Discussion

Selenium has been known for its beneficial function. In recent years, accumulated evidence has indicated that much of the beneficial effect of selenium is attributed to its antioxidant nature and being a functional component of selenoproteins including selenoenzymes. Selenium supplementation has been reported to have positive effect in various diseases and stress conditions including Keshan disease, thyroid hormone metabolism, excitotoxicity, neurodegenerative diseases and cancer [29]. Lifelong low selenium level is associated with compromised cognitive function [30]. In the present study, we investigated the effect of selenium pretreatment on glutamate toxicity, hypoxic and ischemic brain injury. Our data show that selenium treatment decreased cell death and improved cell viability from glutamate toxicity and hypoxia. The positive effect of selenium is mediated through lowering ROS production/accumulation, and preserving mitochondrial membrane potential and mitochondrial functional performance. These *in vitro* effects of selenium were positively translated to *in vivo* stroke model. Therefore, selenium pretreatment decreased infarct volume, reduced oxidative DNA damage and showed neuroprotection. Additionally, we detected the increased protein levels of mitochondrial biogenesis regulators NRF1 and PGC-1α whereas autophagy modulators Beclin 1 and LC3 significantly decreased following selenium pretreatment.

Cerebral ischemia leads to severe structural and functional loss of neurons in the affected region of the brain. Our study with NeuN and Fluoro-Jade B staining revealed that under ischemic conditions selenium pretreatment reduced neurodegeneration and neuronal loss, thereby preserving neuronal integrity. Moreover, selenium pretreatment markedly reduced DNA oxidation following cerebral ischemia. Available evidence suggests that ischemia/reperfusion induces mitochondrial dysfunction by enhancing ROS generation, leading to the damage of intracellular proteins, lipids, and DNA [2,31,32]. Presently, we observed that selenium pretreatment significantly reduced ROS production in our *in vitro* model of glutamate toxicity [33] and hypoxia [18], which may be associated with the selenium-induced increase in activities of antioxidant enzymes [12,34]. Likewise, our *in vitro* study has shown that selenium pretreatment protects mitochondrial functional performance by preserving mitochondrial membrane potential and the activities of mitochondrial complexes. These results are in direct correlation with the available evidence that indicates the important role of selenium in regulating ATP production and activities of mitochondrial respiratory chain complexes [16,35,36]. Thus, it seems most likely that selenium protects mitochondrial function and inhibits mitochondria-initiated cell death pathway, which thereby improves neuro-survival [12]. Additionally, reduction in DNA oxidation observed presently may be attributed to the anti-oxidative nature of selenium, which under these conditions significantly reduced neuronal loss as compared to normal animals.

Reported evidence suggests that selenium accumulates mainly in mitochondria and nuclei in rat *in vivo* pretreatment trials [37] and also present as the crucial component in selenoproteins. Deficiency of selenium or mutation in selenoenzymes such as glutathione peroxidase (GPx) decreases the expression or activity of these enzymes [9] and may exacerbate neuronal loss, whereas selenium pretreatment-dependent increase in activity or overexpression of selenoenzymes ameliorates outcome during endogenous or exogenous stimuli, trauma and other neurodegenerative conditions including cerebral stroke [10-14].

Selenium has been shown to protect mitochondrial function by upregulating mitochondrial biogenesis [12,37,38]. Cerebral ischemia on the other hand is known to damages mitochondria, increases ROS production and impairs ATP generation. Neurons in these conditions initiate adaptive response through activation of mitochondrial biogenesis [39]. Therefore, in the present study we analyzed the protein markers of mitochondrial biogenesis (PGC-1α and NRF1). We found the increased protein levels of PGC-1α and NRF1 at 5- and/or 24 h of recirculation. These results are in accordance with the reports that showed marked increase in mitochondrial DNA content, mitochondrial proteins and numbers, and mRNA levels of NRF1 and Tfam after hypoxia and ischemia [40,41]. Interestingly, selenium pretreatment increased the protein levels of PGC-1α and NRF1 at basal level and increased further after cerebral ischemia and recirculation as compared to respective control. Previous reports have also shown that selenite supplementation increased the level of NRF1, which clearly support our result that mitochondrial biogenesis could be modulated by selenite application [42]. In a parallel study (Li and collegues, unpublished data), we have observed that selenium increases mitochondrial biogenesis markers and mitochondrial proteins cytochrome *c* and COX IV under normal culture condition, inhibits mitochondrial fission induced by glutamate exposure, and induces phosphorylations of Akt, PKA and CREB, transcription factors that are known to activate mitochondrial biogenesis. Thus, selenium induced mitochondrial biogenesis could be an important strategy to improve mitochondrial function in various stress conditions including neurodegenerative diseases. In fact, our *in vitro* hypoxic study demonstrated that selenium increased mitochondrial oxidative phosphorylation and ameliorated the hypoxia-induced suppression to respiratory complex activity.

Autophagy is a major catabolic contributor to degrade and recycle macromolecules and organelles. We and others have found that autophagy markers Beclin 1 and LC3-II were increased following cerebral ischemia [25,43]. Autophagy activation following cerebral ischemia is a process to recycle injured cells or a process responsible for cell demise [43-45]. Interestingly, selenium pretreatment reduced the protein level of Beclin 1 and LC3-II cleavage. It has been reported that ROS is a major factor involved in activation of autophagy [46,47] and selenium lowers ROS production and prevents mitochondrial dysfunction [34,48]. Therefore, it is possible that selenium preserve mitochondrial function, lowers ROS production, reduces autophagy and thereby provides neuroprotection.

## Conclusions

Our results indicate that selenium pretreatment within the physiological dosage attenuates glutamate toxicity and hypoxia-induced cell damage *in vitro* and ameliorates ischemic brain injury *in vivo*. The selenium-dependant neuroprotective effect may be mediated through lowering ROS production, preventing DNA oxidation, preserving mitochondrial membrane potential and mitochondrial functional performance, activating mechanisms that stimulate mitochondrial biogenesis and inhibiting autophagy activation. These results therefore highlight the promising therapeutic potential of selenium against glutamate toxicity, hypoxic and ischemic brain damage.

## Competing interests

The authors declare that they have no competing interests.

## Authors’ contribution

SLM carried out in vivo ischemic stroke experiments, measured mitochondrial respiration, performed data analyses and drafted the manuscript. SK carried out *in vitro* glutamate experiment and ROS measurement. NM performed cell cultures and participated in mitochondrial complex activity measurement. PAL conceived, developed and oversaw the study and contributed to the writing of this manuscript. All authors participated the data evaluation, interpretation and approved the final manuscript.
